# What are the differences in paraspinal muscle morphometry among degenerative spondylolisthesis patients, isthmic spondylolisthesis patients, and healthy individuals? A propensity score matching analysis

**DOI:** 10.1186/s12891-024-07532-9

**Published:** 2024-05-24

**Authors:** Xing-Bin Li, Lu Wang, Qian Deng, Bang Wang, Zhao-Rui Wang, Chun-Ming Zhao, Xiao-Jing Li, Ai-Bing Huang

**Affiliations:** 1 Postgraduate School, Dalian Medical University, Dalian, Liaoning 116000 China; 2 Department of Spine Surgery, Taizhou School of Clinical Medicine, The Affiliated Taizhou People’s Hospital of Nanjing Medical University, Nanjing Medical University, Taizhou, Jiangsu 225300 China; 3 Postgraduate School, Nanjing University of Chinese Medicine, Nanjing, Jiangsu 210000 China; 4 Department of Anesthesiology, Taizhou School of Clinical Medicine, The Affiliated Taizhou People’s Hospital of Nanjing Medical University, Nanjing Medical University, Taizhou, Jiangsu 225300 China

**Keywords:** Lumbar, Degenerative spondylolisthesis, Isthmic spondylolisthesis, Paraspinal muscles

## Abstract

**Purpose:**

To compare the morphometry of paraspinal muscles in patients with degenerative spondylolisthesis (DS), isthmic spondylolisthesis (IS), and healthy individuals.

**Methods:**

Thirty-seven pairs of DS patients were selected using propensity score matching with IS patients, while 37 healthy individuals matched for age, sex, and BMI were selected as controls. The relative cross-sectional area (rCSA), and relative functional cross-sectional area (rfCSA) of paraspinal muscles were measured, and the degree of fatty infiltration (FI) was calculated. Based on occupational differences, the patients were also divided into worker and farmer groups, and the same measurements were taken on them.

**Results:**

At the L3/L4 level, the multifidus (MF) FI was greater in the DS and IS groups than in the control group, the erector spinae (ES) rfCSA was higher in the IS group than in the DS and control groups. At the L4/L5 level, MF rfCSA was smaller in the DS and IS groups than in the control group; ES rfCSA was higher in the IS group than in the DS and control groups. At the L5/S1 level, MF rfCSA was smaller in the DS and IS groups than in the control group; ES rfCSA was higher in the IS group than in the DS group. At the L3/L4, L4/L5 level, MF rfCSA were higher in the worker group than in the farmer group (*p* < 0.05).

**Conclusion:**

The morphological changes in paraspinal muscles in patients with DS were dominated by selective atrophy of the MF, while in patients with IS, the morphological changes in paraspinal muscle showed selective atrophy of the MF accompanied by compensatory hypertrophy of the ES. The surgeon should consider the morphological differences in paraspinal muscle between different types of lumbar spondylolisthesis when establishing the appropriate surgical program.

## Introduction

Based on the Wiltse-Newman classification system, spondylolisthesis is divided into five types [1]. The most common types are isthmic spondylolisthesis (IS) and degenerative spondylolisthesis (DS) [2]. Different pathogenic mechanisms exist for each type but ultimately result in the development of vertebral instability. Paraspinal muscles, such as the erector spinae (ES), multifidus (MF), and psoas major (PS), directly affect the segmental stability and functional activity of the lumbar spine. Evaluation of the morphology of paraspinal muscles in patients with different types of lumbar spondylolisthesis could contribute to unravelling the biological processes involved in the development of lumbar spondylolisthesis. By recognizing the different compensatory mechanisms that may exist in patients with different types of lumbar spondylolisthesis, important clues can be provided for the development of targeted rehabilitation and treatment plans, leading to individualised treatment strategies. Such personalised treatment plans will be more likely to improve patient outcomes and quality of life.

Previous studies have explored the pathological alterations of the paraspinal muscles in lumbar spondylolisthesis. Several studies have demonstrated that MF in patients with DS has a high level of fatty infiltration (FI) [3, 4]. It has also been shown that patients with IS have atrophy of the MF muscle in combination with hypertrophy of the ES muscle compared to normative controls [5]. However, most studies have only addressed morphological alterations in the paraspinal muscles of patients with just one type of lumbar spondylolisthesis. Fewer studies have compared the differences in paraspinal muscles between DS and IS. In addition, previous studies [6, 7] have shown that differences in baseline data such as gender, age, and BMI, may lead to confounding effects of changes in paravertebral muscles. The propensity score matching method serves as a widely used statistical method. Its strength lies in its ability to effectively control the effects of confounding factors thereby improving the internal validity of the study. Therefore, the objective of this study was to explore the differences in the cross-sectional area (CSA) and degree of fatty infiltration (FI) of the paraspinal muscles between the DS and IS groups as well as the normal control group by means of the propensity score matching method.

## Method

### General Information

A retrospective analysis of data from patients diagnosed with lumbar spondylolisthesis who underwent posterior lumbar interbody fusion at our institution from March 2018 to May 2022 was reviewed. Information including sex, age, occupation, height, and weight of patients was recorded. The inclusion criteria were (1) age between 35 and 80 years, (2) diagnosis of L4-L5 or L5-S1 spondylolisthesis with less than 50% slip, and (3) complete preoperative imaging, including lumbar spine X-ray and MRI. The exclusion criteria were (1) spinal deformity, lumbar spine infection, and tumors, (2) previous history of lumbar spine fracture and spine surgery, (3) multi-segmental spondylolisthesis, (4) “non-isthmic (type IIA) or non-degenerative spondylolisthesis, (5) low-quality MRI images of the lumbar spine and (6) previous treatment with local steroid injections, cryoablation or radiofrequency ablation techniques.

**Fig. 1:**
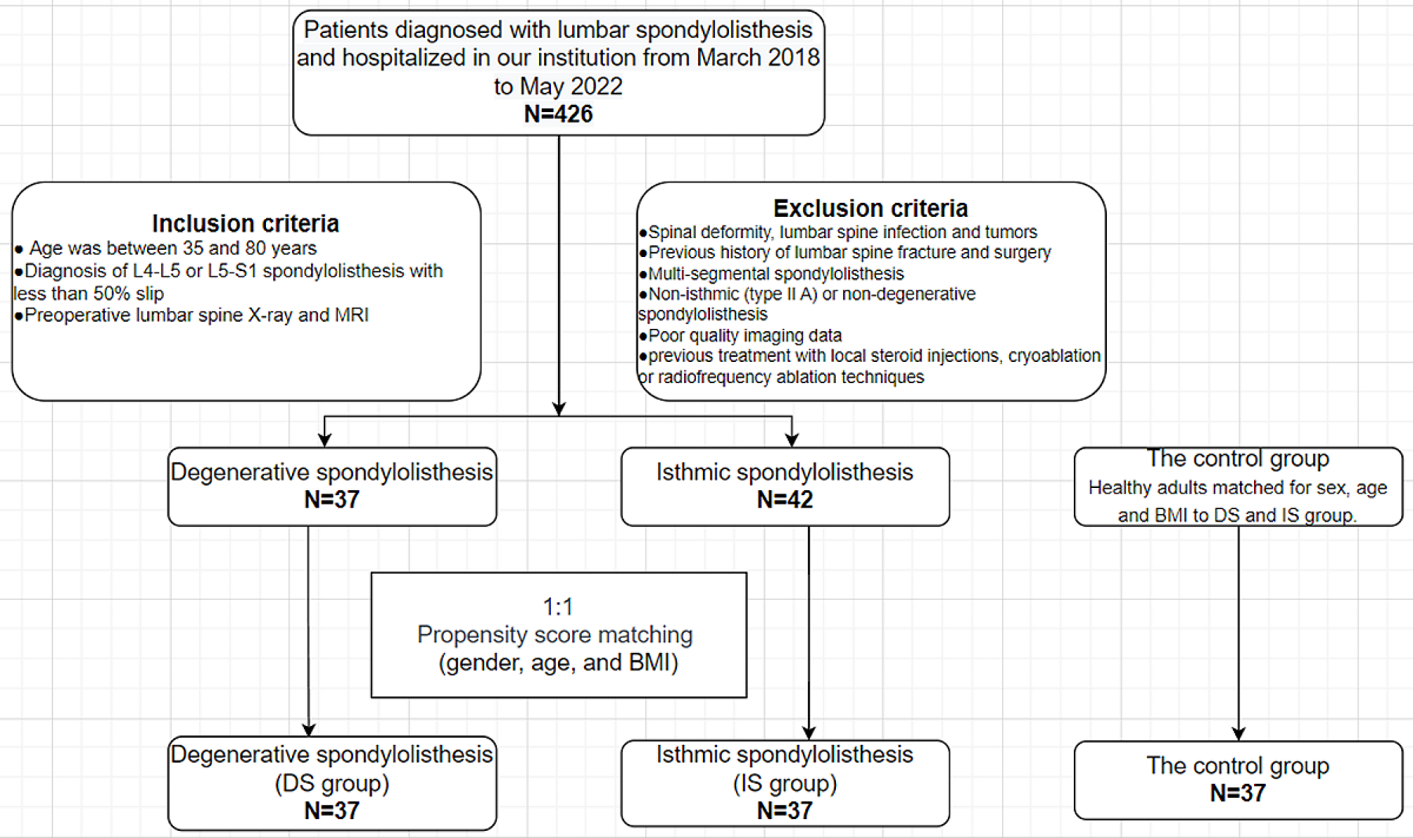
Flowchart of the study participant enrollment

Based on the above inclusion and exclusion criteria, 37 patients with DS and 42 patients with IS were ultimately included in this study. By using propensity score matching, we matched the two groups of patients to create the DS group and an IS group. For the control group, 37 age-, sex- and BMI-matched healthy adults who attended our outpatient clinic and showed no abnormalities on lumbar MRI were recruited (Fig. 1). This study was approved by the ethics committee of our hospital.

### Radiological measurements

**Fig. 2:**
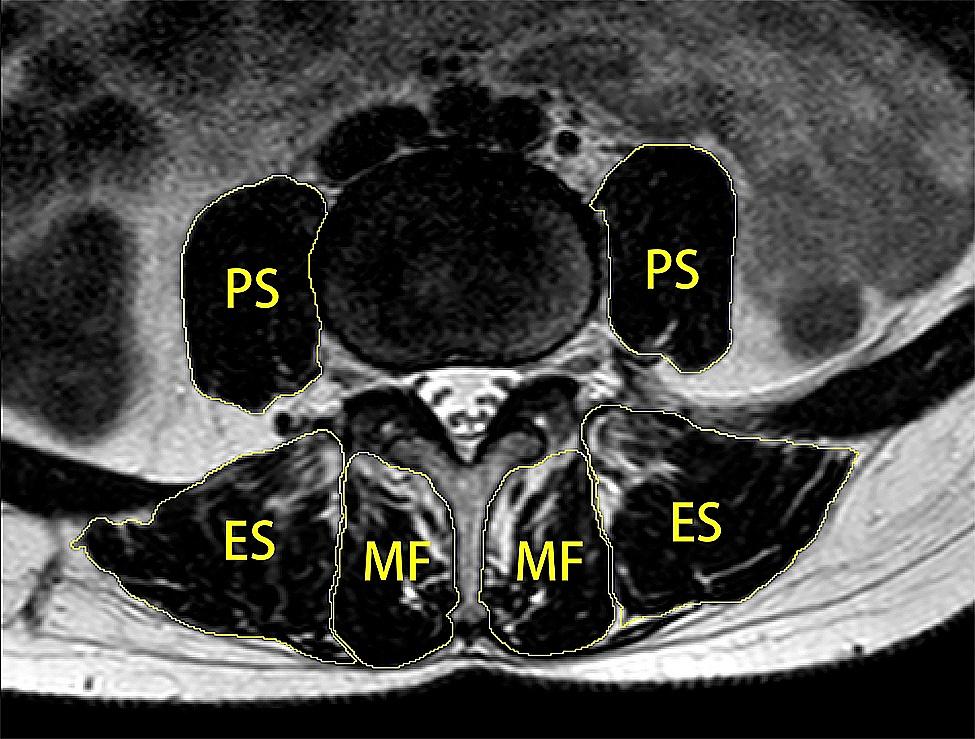
Measurement of the cross-sectional area (CSA) of the psoas major (PS), multifidus (MF), and erector spinae (ES) by creating ROIs

**Fig. 3:**
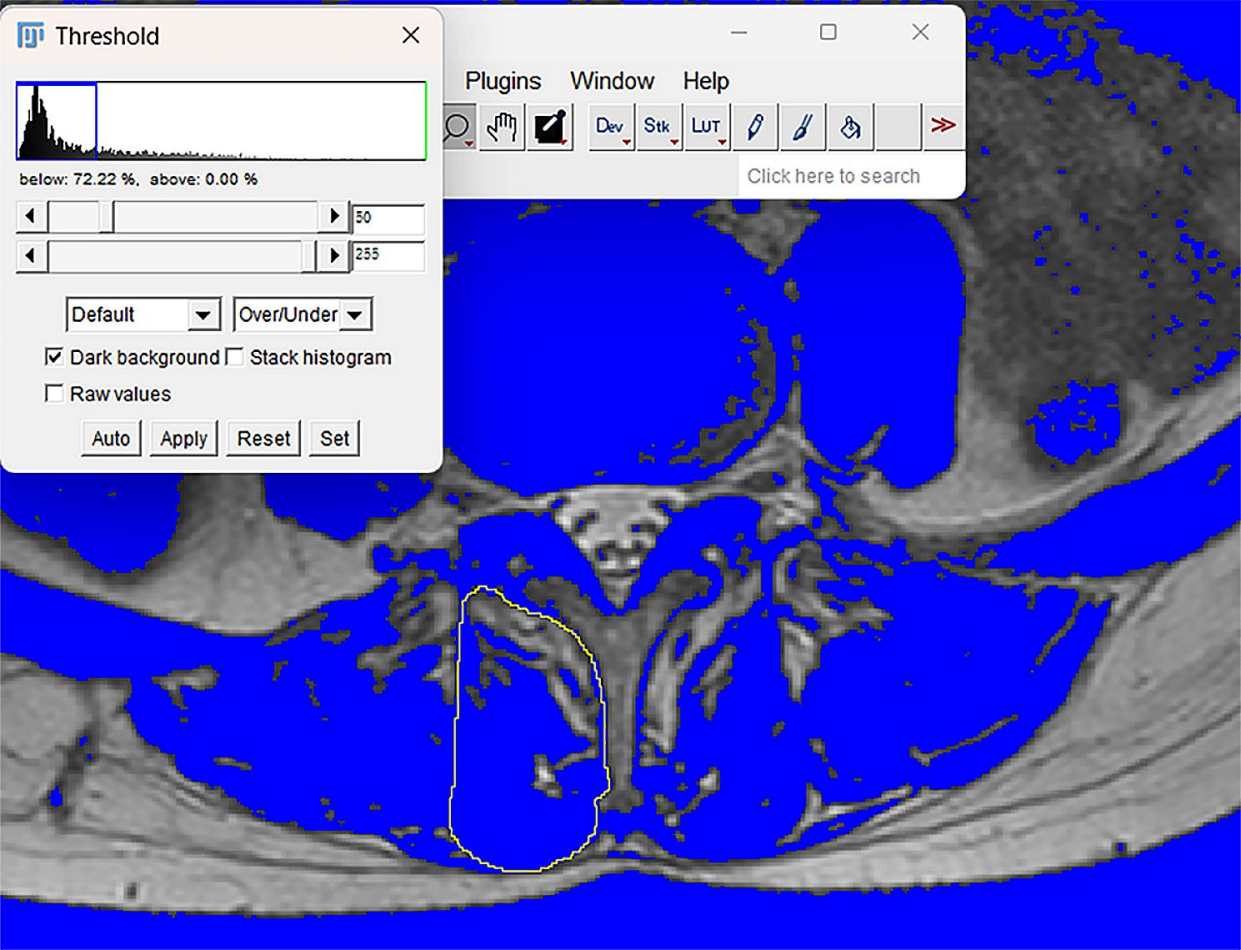
Measurement of multifidus (MF) fatty cross-sectional area (beyond the blue pixels) by the thresholding technique with ImageJ software

The T2-weighted cross-sectional MRI images were derived from a picture archiving and communication system (PACS). Bilateral measurements of all muscles at the intermediate images of each intervertebral disc location at L3 to S1, including the PS, ES, and MF, were performed using ImageJ software (version 1.8.0; National Institutes of Health, USA). The measurement of muscle CSA was ascertained by manually sketching the fascial boundaries of the muscles as described above. (Fig. 2) Relative cross-sectional area (rCSA) was defined as the ratio of muscle CSA to the corresponding vertebral body CSA to diminish the effect of individual body size, weight, and height on muscle CSA [8]. The fatty CSA in the muscle was calculated using a thresholding technique. Fat tissue pixels in the paraspinal muscle ROI were distinguished by the appropriate threshold gray value used [9]. The area occupied by the number of fat tissue pixels in each paraspinal muscle ROI was its fatty CSA [10]. The measurement method is shown in Fig. 3 The functional cross-sectional area (fCSA) was derived by calculating the difference between the CSA of the muscle and the fatty CSA within the muscle, which represented the area of lean muscle. The relative fCSA (rfCSA) was simultaneously calculated, which was defined as the ratio of muscle fCSA to corresponding vertebral body CSA. FI, defined as the ratio of fatty CSA of the muscle to its CSA, was used to demonstrate the regression of the paraspinal muscles. The flexor-extensor CSA ratio was also measured, which was defined as the ratio of the CSA of the flexor muscle (such as PS) to the CSA of the extensor muscles (both ES and MF).

### Sample size and data analysis

The sample size for this study was determined by a power analysis that incorporated data from previous literature measuring paraspinal muscles. For the calculation of the sample size, we used data from the literature [11]: the mean of the relative psoas major cross-sectional area was 1.13 with a standard deviation of 0.31 for the isthmic spondylolisthesis group and 1.46 with a standard deviation of 0.36 for the control group. The power value was taken to be 0.90. The calculations yielded a minimum of 23 individuals should be enrolled in each group.

All statistical analyses were performed using SPSS software (version 26.0; IBM Corp, USA). Propensity scores were evaluated using multiple logistic regression analysis with sex, age, and BMI as covariates. The propensity score matching method was used to match DS patients with IS patients in a 1:1 ratio (caliper 0.2). Quantitative normally distributed data are expressed as the mean ± standard deviation, and quantitative nonnormally distributed data are expressed as the median and interquartile range. Categorical variables were assessed using the chi-square test, while continuous variables (e.g., paraspinal muscle parameters) were evaluated using one-way ANOVA or the Kruskal-Wallis H test, from which differences in radiologic parameters between the DS, IS and control groups were assessed. The statistical significance level was set at *P* < 0.05. The intra- and interobserver agreement were assessed by the intraclass correlation coefficient (ICC). For intra-observer repeatability, all the measurements were repeated by the primary observer for 20 randomly selected cases. For inter-observer repeatability, all the measurements were repeated by an independent secondary observer for 20 randomly selected cases.

## Results

Using propensity score matching, we matched 37 pairs of DS patients with IS patients. The baseline characteristics of the patients in each group are shown in Table 1 There were 14 males and 23 females in the DS group, with an average patient age of 60.46 ± 8.36 years. There were 17 males and 20 females in the IS group, with an average patient age of 57.41 ± 7.31 years. In the control group, there were 16 males and 21 females, with an average age of 57.16 ± 8.04 years, and there were no statistically significant differences in age, sex, occupation or BMI among the three groups. There were 16 workers and 21 farmers in the DS group and 13 workers and 24 farmers in the IS group(*p* = 0.48). The intra- and interobserver ICC values for the 11 parameters are summarized in Table 2.

Paravertebral muscle parameters at different levels between each group are shown in Table 3. At the L3/L4 level, MF rCSA and ES rCSA were significantly higher in the IS group than in the DS and control groups (*p* < 0.01); ES rfCSA was higher in the IS group than in the DS and control groups (*p* < 0.05); MF FI was lower in the control group than in the IS and DS groups (*p* < 0.05); MF fatty CSA was significantly lower in the control group than in the IS and DS groups (*p* < 0.01); the flexor-extensor CSA ratio was significantly higher in the control group than in the IS and DS groups. (*p* < 0.01)

At the L4/L5 level, ES rCSA was significantly higher in the IS group than in the DS and control groups (*p* < 0.01); ES rfCSA was higher in the IS group than in the DS and control groups (*p* < 0.05); MF fatty CSA was significantly higher in the IS group than in the control group (*p* < 0.01); MF FI was higher in the IS group than in the control group (*p* < 0.05); MF rfCSA and the flexor-extensor CSA ratio were higher in the control group than in the IS and DS groups.(*p* < 0.05).

At the L5/S1 level, MF rCSA and MF rfCSA were significantly higher in the control group than in the IS and DS groups (*p* < 0.01); MF rCSA was significantly higher in the DS group than in the IS groups (*p* < 0.01); ES rfCSA was higher in the IS group than in the control group (*p* < 0.05); and ES FI was significantly smaller in the IS group than in the DS and control groups. (*p* < 0.01)

In addition, patients in the DS and IS groups were regrouped into worker and farmer groups according to occupation. The baseline characteristics of the patients in each group are shown in Table 4 There were 11 males and 18 females in the worker group, with an average patient age of 55(15) years. There were 21 males and 24 females in the farmers group, with an average patient age of 59(11) years. There were no statistically significant differences in age, sex, or BMI between the two groups.

Paravertebral muscle parameters at different levels between worker and farmer groups are shown in Table 5. At the L3/L4 level, MF rfCSA were higher in the worker group than in the farmer group (*p* < 0.05); and MF FI was smaller in the worker group than in the farmer group (*p* < 0.05).

At the L4/L5 level, MF rfCSA were higher in the worker group than in the farmer group (*p* < 0.05).

**Table 1:** Baseline characteristics for the DS and IS groups after propensity score matching versus the control group

	Propensity score matched data					
	DS group(*n* = 37)	IS group(*n* = 37)	*P* value		Control group(*n* = 37)	*P* value
Age (years)	60.46 ± 8.36	57.41 ± 7.31	0.099		57.16 ± 8.04	0.141
Gender (M/F)	14/23	17/20	0.480		16/21	0.772
BMI (kg/m^2^)	24.45 ± 2.24	24.11 ± 3.18	0.598		23.21 ± 3.42	0.191

DS: degenerative spondylolisthesis; IS: isthmic spondylolisthesis; BMI: body mass index

**Table 2:** Coefficients of repeatability for interobserver and intraobserver measurement repeatability

	Intraobserver	Interobserver
MF rCSA	0.945	0.926
MF rfCSA	0.948	0.840
ES rCSA	0.980	0.972
ES rfCSA	0.930	0.892
PS CSA (mm^2^)	0.989	0.999
MF Fatty CSA (mm^2^)	0.965	0.720
MF FI	0.959	0.798
ES Fatty CSA (mm^2^)	0.916	0.819
ES FI	0.884	0.717
Flexor-extensor CSA ratio	0.976	0.987

MF: multifidus; ES: erector spinae; PS: psoas major; CSA: cross-sectional area; rCSA: relative cross-sectional area; rfCSA: relative functional cross-sectional area; FI: fatty infiltration; Flexor-extensor CSA ratio: the ratio of the PS CSA to the sum of MF CSA and ES CSA

**Table 3:** Paravertebral muscle parameters at different levels in the three groups of patients

Level	Group	MF rCSA	MF rfCSA	ES rCSA	ES rfCSA	PS CSA (mm^2^)	MF Fatty CSA (mm^2^)	MF FI	ES Fatty CSA (mm^2^)	ES FI	Flexor-extensor CSA ratio
L3/L4	DS group	0.66 ± 0.15	0.42 ± 0.12	1.78 ± 0.31	1.39(0.44)	1366.38(568.09)	439.87(200.73)	0.36(0.17)	844.35(415.09)	0.23(0.14)	0.32(0.10)
	IS group	0.74 ± 0.13	0.45 ± 0.12	2.03 ± 0.43	1.47(0.53)	1614.8(887.7)	464.44(309.13)	0.36(0.20)	723.54(404.67)	0.20(0.13)	0.33(0.12)
	Control group	0.63 ± 0.13	0.44 ± 0.13	1.79 ± 0.37	1.32(0.52)	1509.83(906.94)	356.06(220.74)	0.28(0.18)	635.14(277.31)	0.20(0.10)	0.38(0.11)
	*P* value	0.005^**^	0.553	0.008^**^	0.022^*^	0.466	0.001^**^	0.013^*^	0.058	0.130	0.006^**^
L4/L5	DS group	0.84 ± 0.18	0.49 ± 0.14	1.44 ± 0.34	1.02 ± 0.33	1996.2(900.26)	662.44 ± 223.75	0.41(0.20)	794.81(461.79)	0.29(0.15)	0.46(0.14)
	IS group	0.93 ± 0.21	0.52 ± 0.18	1.70 ± 0.42	1.23 ± 0.35	2145.41(1186.95)	764.74 ± 264.96	0.42(0.21)	799.42(352.93)	0.24(0.15)	0.44(0.18)
	Control group	0.92 ± 0.14	0.60 ± 0.16	1.47 ± 0.29	1.04 ± 0.26	2146.26(1133.95)	578.63 ± 187.95	0.33(0.15)	745.24(382.51)	0.27(0.15)	0.50(0.18)
	*P* value	0.058	0.018^*^	0.004^**^	0.010^*^	0.500	0.003^**^	0.013^*^	0.446	0.601	0.014^*^
L5/S1	DS group	1.07 ± 0.26	0.63 ± 0.22	0.82(0.54)	0.41(0.33)	1952.91(1098.78)	752.53 ± 209.35	0.40(0.18)	712.17(410.07)	0.44(0.20)	0.59(0.27)
	IS group	0.94 ± 0.26	0.55 ± 0.19	1.05(0.76)	0.76(0.56)	2089.14(1101.93)	767.43 ± 279.39	0.42(0.21)	680.65(499.16)	0.33(0.20)	0.56(0.21)
	Control group	1.26 ± 0.26	0.77 ± 0.23	0.98(0.69)	0.53(0.45)	2203.89(986.41)	789.11 ± 242.61	0.38(0.17)	772.7(322.81)	0.48(0.19)	0.57(0.24)
	*P* value	0.000^**^	0.000^**^	0.073	0.011^*^	0.338	0.813	0.721	0.169	0.000^**^	0.910

DS: degenerative spondylolisthesis; IS: isthmic spondylolisthesis; MF: multifidus; ES: erector spinae; PS: psoas major; CSA: cross-sectional area; rCSA: relative cross-sectional area; rfCSA: relative functional cross-sectional area; FI: fatty infiltration; Flexor-extensor CSA ratio: the ratio of the PS CSA to the sum of MF CSA and ES CSA. * *P*<0.05: ** *P*<0.01

**Table 4:** Baseline characteristics for the worker and farmer groups

	Worker group(*n* = 29)	Farmer group(*n* = 45)	*P* value
Age (years)	55(15)	59(11)	0.224
Gender (M/F)	11/18	21/24	0.459
BMI (kg/m^2^)	24.61 ± 3.29	24.06 ± 2.33	0.404

BMI: body mass index; M: male; F: female

**Table 5:** Paravertebral muscle parameters at different levels between worker and farmer groups

	Level	Worker group	Farmer group	*P* value
MF rCSA	L3/L4	0.71 ± 0.15	0.69 ± 0.13	0.656
	L4/L5	0.92 ± 0.24	0.86 ± 0.18	0.189
MF rfCSA	L3/L4	0.47 ± 0.13	0.41 ± 0.11	0.037^*^
	L4/L5	0.55 ± 0.19	0.47 ± 0.13	0.038^*^
ES rCSA	L3/L4	1.88 ± 0.48	1.92 ± 0.34	0.717
	L4/L5	1.55 ± 0.43	1.58 ± 0.39	0.724
ES rfCSA	L3/L4	1.44 ± 0.48	1.46 ± 0.32	0.822
	L4/L5	1.03(0.56)	1.10(0.48)	0.67
PS CSA (mm2)	L3/L4	1365.21(764.09)	1437.03(746.29)	0.769
	L4/L5	1945.69(1116.46)	2145.41(971.66)	0.385
MF Fatty CSA (mm2)	L3/L4	422.44(174.66)	485.76(289.91)	0.102
	L4/L5	616.20(309.78)	726.79(345.47)	0.155
MF FI	L3/L4	0.34 ± 0.11	0.40 ± 0.13	0.048^*^
	L4/L5	0.40 ± 0.13	0.44 ± 0.14	0.176
ES Fatty CSA (mm2)	L3/L4	784.63 ± 254.08	813.18 ± 336.27	0.697
	L4/L5	802.40(476.33)	794.81(379.59)	0.934
ES FI	L3/L4	0.24 ± 0.08	0.24 ± 0.10	0.739
	L4/L5	0.30 ± 0.11	0.28 ± 0.12	0.434

MF: multifidus; ES: erector spinae; PS: psoas major; CSA: cross-sectional area; rCSA: relative cross-sectional area; rfCSA: relative functional cross-sectional area; FI: fatty infiltration. * *P*<0.05: ** *P*<0.01

## Discussion

The paraspinal muscles are essential in the stabilization of the lumbar spine, especially in patients with lumbar spondylolisthesis. The degree of FI of the paraspinal muscles is a useful indicator of paraspinal muscle quality [12]. Previous studies have shown varying degrees of muscle density and FI ratios in the paraspinal muscles [13]. In a recent study, Li et al. [11] performed a comparative study of the CSA and the FI degree of the paraspinal muscles in patients with IS and DS, and they demonstrated that patients with IS and DLS showed varying degrees of degeneration. However, the heterogeneity of the baseline data in the study made it impossible to draw any conclusive results. Therefore, to improve comparability, we used propensity score matching to minimize differences in baseline data between the IS and DS groups. In addition, the method of quantitatively measuring the morphometry of the paraspinal muscle in the present study was proposed by previous studies [14], which facilitated further comparison of the morphological differences in the paraspinal muscles among the groups.

The MF is the largest and medial-most group of paraspinal muscles [15], and it performs independent biomechanical actions in each segment [16]. The degree of FI and the size of the CSA are important indicators to assess the quality of the paraspinal muscles [12]. By considering the effect of fatty tissue contained in the muscle on the measurement of the CSA of the paraspinal muscle, we analyzed the MF rfCSA among the three groups. In the present study, at the L4/L5 and L5/S1 levels, MF rfCSA was less in both the IS and DS groups than in the control group, which was consistent with previous findings. In a CT study, atrophy of the MF was selective, as no significant change in CSA was observed in either PS or ES compared to controls [17]. Thakar et al. [5] assessed the CSA of the paraspinal muscles of the lumbar spine in 120 adult patients with IS, which suggested that the CSA of the MF was significantly lower in patients with IS than in normal population. These findings suggest that MF atrophy was present in both the DS and IS groups. A previous study showed that the fat replacement of muscle might not significantly change its CSA while altering its function [18]. To better evaluate the quality of the paraspinal muscles, an assessment of the degree of FI of the paraspinal muscles was also essential. In the present study, the MF FI was much higher in the IS groups than in the control group. At the L3/L4 level, the DS group also had a higher MF FI than the control group. This result was consistent with previous studies on FI of the paraspinal muscles in patients with lumbar spondylolisthesis. By evaluating paraspinal muscle density by computer tomography (CT), Leonid Kalichman et al. [6] discovered a significant correlation between MF density at the L4 segment and lumbar spondylolisthesis. Eun Taek Lee et al. [3] found a higher degree of MF FI in patients with DS than in patients with chronic radiculopathy. Li et al. [11] showed that both DS patients and IS patients had varying degrees of MF FI. Together with the results of the present study, these findings demonstrated that regeneration of the MF occurred in both IS and DS, which may be manifested in the form of reduced rfCSA and more severe FI.

The deeper fibers of MF in the paraspinal muscles contain more type I (slow-twitch) muscle fibers [5], and these anti-fatigue fibers are adapted for low-load tension activities and have a greater susceptibility to immobilization or pain than type II (fast-twitch) muscle fibers [19]. This may account for the selective atrophy of the MF in patients with DS and IS. The segmental hypermobility that often occurs in patients with lumbar spondylolisthesis may lead to MF denervation and the development of MF atrophy [20]. Franke et al. revealed that when the nerve roots innervating the MF are compressed, they are prone to MF denervation, further leading to MF atrophy [21].

The ES consists of the longissimus and iliocostalis, innervated by the intermediate and lateral branches of the dorsal ramus [22, 23]. Thakar et al. [5] compared the CSA of the paraspinal muscles between IS patients and the normal population and found that patients with IS experienced selective atrophy of the MF and compensatory hypertrophy of the ES. It has also been shown that patients with DS suffer from a higher incidence of paraspinal muscle hypertrophy than normal individuals [24]. However, few studies have reported differences between IS and DS patients regarding ES. In a comparative study, Li et al. [11] showed compensatory hypertrophy of the ES in patients with IS, while patients with DS showed atrophy of the ES, which is inconsistent with previous studies. They hypothesized that the reason for this phenomenon was due to the relatively advanced age of the DS patient, that the compensatory capacity of ES was limited, or that ES entered the decompensated phase. In the present study, ES rfCSA was significantly greater in the IS group than in the DS and control groups, whereas ES rfCSA was not significantly different in DS patients compared to controls. These results confirmed and broadened the findings of previous studies. We can carefully conclude that IS patients possessed a larger volume of ES than DS patients and that there was compensatory hypertrophy of the ES.

The mechanisms of disease progression for IS and DS were distinct. The anatomy of the vertebrae’s pedicles and facet joints, the paraspinal muscle groups, and the presence of intervertebral discs are regarded as crucial factors in maintaining the stability of the spine. The slippage of the vertebrae could be accounted for by a considerable anterior shear force on the lumbosacral junction [25]. The pedicles formed a bony bridge that transmitted forces and maintained segmental stability. In DS, intact pedicles could resist vertebral shearing forces and prevent further its slippage [26]. In IS, the interarticular defect resulted in an incomplete vertebral arch ring and required additional compensatory mechanisms to retard vertebral slippage. We suggest that the paraspinal muscles, particularly the ES, play a compensatory role in the disease progression of IS. The MF of the paraspinal muscle group, close to the center of rotation of the lumbar joint, plays a major role in controlling intervertebral motion [27]. In contrast to the MF, the ES lies lateral to the MF controls spinal motion, and maintains spinal balance by gradually lengthening its fibers [4]. In the present study, we found that the ES rfCSA was significantly greater in patients with IS than in those with DS. This finding supported our view above. Therefore, the paraspinal muscles of IS, particularly ES, underwent compensatory hypertrophy as a self-protective mechanism to maintain spinal stability. In contrast, the compensatory mechanism of the paraspinal muscles in DS was not significant.

Previous studies have [28] indicated that laborious work had an impact on low back muscle mass. Taking into account the variations in patients’ occupational backgrounds, we categorized the participants into worker and farmer groups based on their occupations. Our findings demonstrated the farmer group had a smaller MF rfCSA and a more severe degree of MF FI compared to the worker group. This finding can be attributed to the prolonged physical labor and repetitive movements involved in farmers’ jobs, which subject their lumbar region to greater stress and consequently promote selective atrophy of the MF. This also highlights the role of the MF in maintaining normal structure and function.

Previous studies have shown that the traditional posterior approach can have a severe impact on the paravertebral muscle groups, which can further impact postoperative recovery [29]. Postoperatively, patients with low back pain developed atrophy of the MF as well as an accumulation of intramuscular fatty tissue [29]. In addition, the local anatomy made the ES and MF vulnerable to injury during conventional PLIF surgery [30]. Procedures with less tissue dissection and traction were effective in reducing disruption to the paraspinal muscles [31, 32]. These findings illuminate the essential role of paraspinal muscles in spinal surgery. Firstly, damage to paraspinal muscles during surgery may lead to postoperative complications and rehabilitation difficulties. Atrophy and fatty infiltration of the muscles may negatively affect patient function, pain levels, and quality of life. Secondly, different types of lumbar spondylolisthesis may induce different changes in muscle morphology, which means that the paraspinal muscles need to be maximized by choosing the appropriate surgical access on an individual basis. By minimizing trauma to the muscles, surgical success, and clinical outcomes can be improved. As discussed above, paraspinal muscles, especially MF and ES, performs an important role in spinal surgery. Comprehending the muscle-clinical connection can help guide the choice of surgical strategy and ultimately enhance patient outcomes and the rehabilitation process.

The present study has several limitations. First, selection bias cannot be completely ruled out due to the retrospective nature of the study. Second, this study was a comparative analysis of the morphology of the paraspinal muscles among different groups, and the causal relationship between atrophy of the paraspinal muscles and lumbar spondylolisthesis was difficult to define.

## Conclusion

The morphological changes in the paraspinal muscles in patients with DS were dominated by selective atrophy of the MF, while in patients with IS, the morphological changes in the paraspinal muscles showed selective atrophy of the MF accompanied by compensatory hypertrophy of the ES. The surgeon should consider the morphological differences in the paraspinal muscles between the different types of lumbar spondylolisthesis when establishing the appropriate surgical program.

## Data Availability

The datasets used or analyzed during the current study are available from the corresponding author upon reasonable request.
